# In-house designed simulation courses versus society-accredited designs by international societies: A comparative analysis

**DOI:** 10.3205/zma001756

**Published:** 2025-06-16

**Authors:** Igor Abramovich, Jakob Beilstein, Eva Kornemann, Joana Berger-Estilita, Torsten Schröder

**Affiliations:** 1 Charité – Universitätsmedizin Berlin, Department for Anaesthesiology and Intensive Care Medicine (CCM/CVK), Berlin, Germany; 2 Berlin Simulation & Training Center (BeST), Berlin, Germany; 3 University of California San Francisco, Department of Anesthesia and Perioperative Care, San Francisco, CA, USA; 4 University of Bern, Institute for Medical Education, Bern, Switzerland; 5 Hirslanden Medical Group, Salemspital, Institute of Anaesthesiology and Intensive Care, Bern, Switzerland

**Keywords:** simulation-based medical education, in-house design, emergency medicine simulation

## Abstract

**Background:**

Simulation-based medical education is increasingly important in postgraduate training, yet the comparative merits of in-house vs. society-accredited courses are still not well understood. This study examined these two approaches in three emergency medicine domains – prehospital, pediatric, and adult – to identify their respective strengths and potential limitations.

**Methods:**

In a retrospective analysis, 1,263 participants from 57 sessions (2019–2023) evaluated six emergency medicine courses (three society-accredited, three in-house). A 25-item Likert-scale survey assessed aspects of course content, delivery, organization, and overall recommendation, alongside demographic questions and free-text comments. Mann-Whitney U tests and Cliff’s Delta were used for statistical comparisons.

**Results:**

Society-accredited courses generally scored higher on guideline adherence, presenter competence, and practical relevance, whereas in-house formats excelled in areas like content scope and communication. Participant specialty, workplace, and training stage influenced ratings. Free-text feedback praised hands-on learning and small-group design but called for earlier material distribution, better logistics, and clearer guidelines.

**Conclusions:**

Both in-house and society-accredited SBME courses exhibit distinct strengths. Adopting best practices from both models, may guide a hybrid approach that optimizes SBME outcomes. However, reliance on self-reported data and a lack of controls for instructor competence or teaching style limit generalizability. Future research should include a broader sample, more rigorous content analysis, longitudinal follow-up, and detailed participant experience data to enhance the depth and applicability of findings.

## Zusammenfassung

**Hintergrund:**

Die simulationsbasierte medizinische Ausbildung gewinnt in der postgradualen Weiterbildung zunehmend an Bedeutung, doch die vergleichenden Vorzüge von internen und von Fachgesellschaften akkreditierten Kursen sind noch nicht ausreichend bekannt. In dieser Studie wurden diese beiden Ansätze in drei Bereichen der Notfallmedizin untersucht – präklinische, pädiatrische und erwachsenen Notfallmedizin, um ihre jeweiligen Stärken und potenziellen Einschränkungen zu ermitteln.

**Verfahren:**

In einer retrospektiven Analyse bewerteten 1263 Teilnehmer aus 57 Sitzungen (2019–2023) sechs Kurse in Notfallmedizin (drei von Fachgesellschaften akkreditierte und drei intern entwickelte Simulationskurse). In einer Umfrage mit 25-Likert-Skala-Items wurden neben demografischen Fragen und Freitextkommentaren auch Aspekte des Kursinhalts, der Durchführung, der Organisation und der allgemeinen Empfehlung bewertet. Für statistische Vergleiche wurden Mann-Whitney-U-Tests und Cliff's Delta verwendet.

**Ergebnisse:**

Von Fachgesellschaften akkreditierte Kurse schnitten im Allgemeinen besser ab, was die Einhaltung von Richtlinien, die Kompetenz der Referenten und die praktische Relevanz betraf, während interne entwickelte Formate in Bereichen wie dem Umfang der Inhalte und der Kommunikation herausragten. Die Bewertungen wurden durch die Fachrichtung, den Arbeitsplatz und die Ausbildungsstufe der Teilnehmer beeinflusst. In den Freitextkommentaren wurden das praktische Lernen und das Kleingruppenkonzept gelobt, aber eine frühere Materialverteilung, eine bessere Logistik und klarere Richtlinien gefordert.

**Schlussfolgerungen:**

Sowohl interne entwickelte Formate als auch von Fachgesellschaften akkreditierte Kurse weisen deutliche Stärken auf. Die Übernahme bewährter Verfahren aus beiden Modellen könnte einen hybriden Ansatz bilden, der die simulationsbasierte medizinische Ausbildung optimiert. Die Abhängigkeit von selbstberichteten Daten und die mangelnde Kontrolle hinsichtlich der Kompetenz der Ausbilder oder des Lehrstils schränken jedoch die Verallgemeinerbarkeit ein. Zukünftige Arbeiten sollten eine breitere Probe, eine strengere Inhaltsanalyse, eine Längsschnitt-Nachverfolgung und detaillierte Daten zur Teilnehmererfahrung einschließen, um die Tiefe und Anwendbarkeit der Ergebnisse zu steigern.

## 1. Introduction

Simulation-Based Medical Education (SBME) has emerged as a transformative force in medical training, offering a dynamic and immersive learning environment that bridges the gap between theoretical knowledge and clinical practice [1]. Over the past few decades, SBME has gained significant traction, becoming an integral component of postgraduate medical education across various specialities worldwide [2], [3], [4]. Medical educators, policymakers, and healthcare professionals have widely recognised its potential to enhance clinical skills, improve patient outcomes, and mitigate medical errors [5]. 

SBME encompasses various simulation modalities, ranging from high-fidelity simulations to standardised patients and procedural part-task trainers [6]. These simulations replicate real-life clinical scenarios, allowing learners to practice clinical skills, decision-making, and teamwork in a safe and controlled environment [7]. As such, SBME has permeated various medical disciplines, including emergency medicine [8], surgery [9], internal medicine [10], and beyond [11]. It has become a cornerstone of postgraduate medical training and extended its reach to pre-graduate medical students and other healthcare practitioners, enriching their educational experiences and preparing them for clinical practice [12].

In recent years, the proliferation of SBME programs on a global scale has been accompanied by a concerted effort to standardise training frameworks and enhance educational outcomes. Medical societies, such as the European Resuscitation Council (ERC) [13], American Heart Association (AHA) [https://cpr.heart.org/en/resources/history-of-cpr] and Resuscitation Council UK [14] have played a pivotal role in meticulously devising SBME guidelines tailored to specific clinical contexts. These guidelines outline curriculum content, format architecture, and faculty preparation, providing a blueprint for high-quality SBME training. Yet, the relevance of comparing different SBME formats – particularly in high-stakes fields such as prehospital, pediatric, and adult emergency medicine – remains paramount for identifying optimal practices and improving patient care.

Despite the prevalence of certified SBME formats, in-house courses – such as those at the Berlin Simulation & Training Center (BeST) – remain distinct. They tailor content to specific specialties and competency levels, emphasizing flexibility and learner-focused design. These courses often employ innovative simulations, customized scenarios, and specialized debriefing methods, reflecting the expertise and resources of the hosting institution [15], [16], [17], [18].

In contrast, society-accredited courses adhere to standardised guidelines and accreditation criteria, providing a structured framework with prescribed curriculum content and instructional materials [19]. These courses undergo rigorous review processes to ensure consistency, quality, and adherence to best practices in SBME [20]. While this approach offers the advantage of recognized standards and uniform delivery across institutions, it may also limit opportunities for individualised learning and local innovation.

This study aims comparatively analyze in-house designed and society-accredited SBME courses within three key areas of emergency medicine – prehospital, pediatric, and adult – focusing on participant evaluations and course design features. By investigating participant evaluations of course content quality, delivery, organization, and overall satisfaction, we aim to clarify each approach’s respective strengths and potential limitations. Our findings intend to inform educators, policymakers, and healthcare stakeholders about how different SBME programs may best serve diverse learner needs, ultimately guiding more effective training initiatives and contributing to improved clinical outcomes.

## 2. Methods

### 2.1. Ethics

Ethics approval was waived by the Charité – Universitätsmedizin Berlin Ethics Committee (EA1/101/24) on 10 May 2024. No identifying data were collected, and all survey information was stored securely with restricted access. The study adhered to the Declaration of Helsinki and relevant Data Protection Acts.

### 2.2. Study design

This retrospective study compared post-course evaluations and free-text responses from in-house (S) vs. society-accredited (A) emergency medicine courses in prehospital (S-PHEM vs. A-PHEM), pediatric (S-PED vs. A-PED), and adult (S-ALS vs. A-ALS) settings (see table 1 (Tab. 1)). The primary focus was to identify differences in content quality, delivery, organization, and overall satisfaction. Detailed learning objectives and key goals for each course are provided in attachment 1 .

All courses were taught by experienced physicians (anesthesiology, intensive care, emergency medicine, or pediatrics). Prehospital formats also included Emergency Medical Services providers. In-house instructors completed a structured training program – observational hospitations and supervised teaching – before becoming full instructors. Society-accredited instructors followed their society’s formal pathway, typically involving high participant performance, an instructor training course, and additional hospitations.

### 2.3. Study outcomes

We compared participant evaluations from in-house (S) vs. society-accredited (A) courses. The primary outcome was the difference in average post-course scores for each category, calculated separately for the courses. The secondary outcome examined the impact of demographic factors (gender, location, specialty, training stage, workplace) on these ratings. Finally, open-ended questions provided additional insights into participants’ perspectives.

### 2.4. Data collection 

Data were collected between January 2019 and December 2023 from participants immediately following each of the six course formats under study. The Berlin Medical Association Course Evaluation Form (English translation in attachment 2 ) was used, consisting of:

- 25 Likert-like scale items, rated from 1 (strongly agree) to 5 (not at all).
- Two free-text questions, prompting participants to describe beneficial aspects of the course and areas needing improvement.
- Demographic questions (gender, practice location, medical specialty, training stage, workplace type).

No incentives were offered. However, completing the evaluation form was part of the mandatory procedure required by the Berlin Medical Chamber for accrediting Continuing Medical Education credits. It typically took about five minutes to finish the form.

The 25 Likert-like items were grouped into four domains:

- Course content (items 1-14): Content relevance, instructor competence, presentation quality, adherence to guidelines, content scope, personal gain/feasibility, and disclosure of conflicts of interest.
- Delivery of content (items 15-19): Methods of content delivery, learning objectives (individual/group work), quality of materials, and discussion opportunities.
- Organisation (items 20-24): Course registration, service/support, moderation, timing, and participant numbers.
- Possible recommendation (item 25)

Additionally, participants were invited to provide free-text remarks (items 26-27).

### 2.5. Data analysis 

All evaluation data were securely stored to protect anonymity, and each of the six course formats underwent both within- and between-group comparisons. Because the Shapiro–Wilk test showed a non-normal distribution, data were analyzed non-parametrically. Mann-Whitney U tests (p<0.05) compared in-house (S) vs. society-accredited (A) courses and demographic factors, with Cliff’s Delta (Δ) quantifying effect sizes. Qualitative feedback was analyzed inductively by three authors: JBE identified recurring themes, IA refined them, and TS validated the final categories.

## 3. Results

### 3.1. Respondents’ characteristics 

A total of 1,263 individuals participated in the six course formats across 57 sessions (January 2019-December 2023). Of these, 868 evaluation forms were returned (68.7% response rate). Table 2 (Tab. 2) illustrates the respondent characteristics and distribution by course type, while table 3 (Tab. 3) provides additional demographic details, including gender, location of practice, medical specialty, role, and workplace of participants.

### 3.2. Comparison of prehospital emergency medicine courses (A-PHEM vs. S-PHEM)

S-PHEM received lower ratings than A-PHEM on “current guidelines” (p=0.038, Δ=-0.123), “selection of contributions” (p=0.039, Δ=-0.176), “scope of content” (p=0.023, Δ=-0.214), and “own level of competence” (p=0.043, Δ=-0.234). It also scored less favorably on “presentation of conflicts of interest” (p=0.001, Δ=-0.351), “processing/mentioning of learning objectives” (p=0.044, Δ=-0.184), “time frame” (p=0.038, Δ=-0.123), “communication/social skills” (p=0.031, Δ=0.059) and “registeration process” (p=0.04, Δ=-0.029).

Within A-PHEM, males rated “critically reflective presentation” higher than females (p=0.029, Δ=-0.196). In S-PHEM, participants outside Berlin viewed “number of participants” more positively (p=0.047, Δ=-0.141).

**Figure 1:**
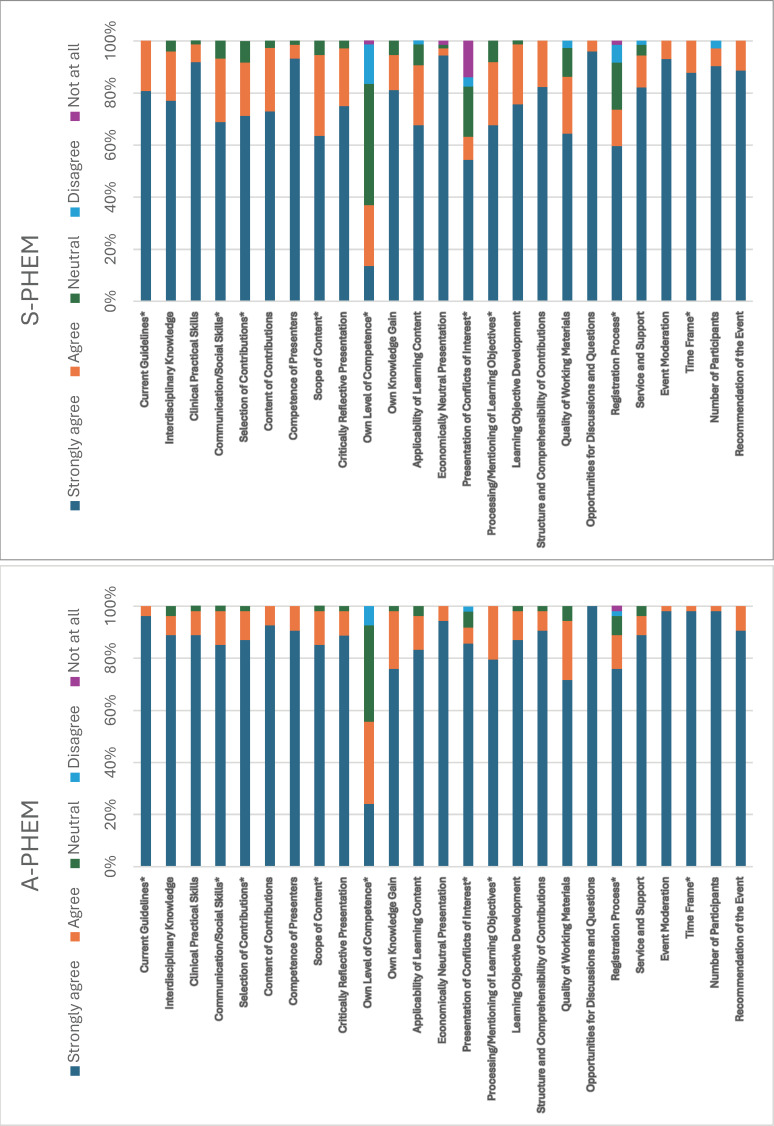
Stacked bar chart showing the percentage distribution of ratings by category for both the accredited prehospital EM course (A-PHEM) and the in-house PHEM course (S-PHEM). Asterisks (*) mark significant differences based on the U test (p<0.05). Full percentage data is provided in attachment 4.

Specialty differences were notable in A-PHEM: non-anesthesiology participants gave higher ratings to “current guidelines,” “communication/social skills,” “content of contributions,” “competence of presenters,” “own knowledge gain,” and “learning objective development alone or in groups” (all p<0.05, Δ range=-0.25 to -1.0). Similar trends appeared in S-PHEM for “interdisciplinary knowledge” (p=0.026, Δ=-0.341), “competence of presenters” (p=0.026, Δ=-0.222), and “learning objective development” (p<0.05 Δ up to -1.0). No significant differences emerged between residents and specialists (p=0.05). Finally, workplace influenced perceptions in A-PHEM, with university participants providing more favorable ratings across multiple aspects (p<0.05, Δ=0.333) (see figure 1 (Fig. 1)).

### 3.3 Comparison of pediatric emergency medicine courses (A-PED vs. S-PED)

**Figure 2:**
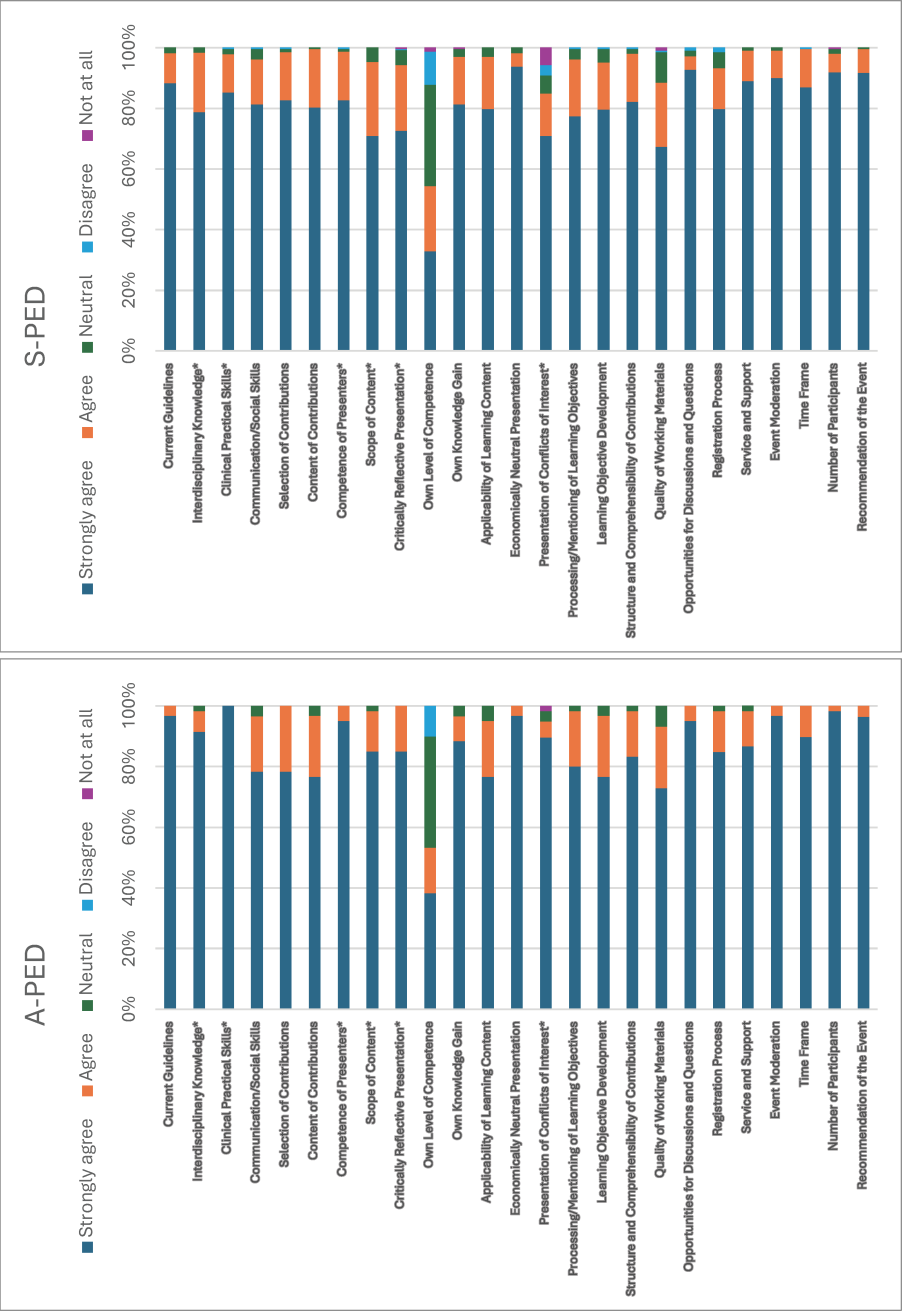
Stacked bar chart showing the percentage distribution of ratings by category for both the accredited pediatric emergence medicine course (A-PED) and the in-house (S-PED). Asterisks (*) mark significant differences based on the U test (p<0.05). Full percentage data is provided in attachment 4.

A-PED scored higher than S-PED on “critically reflective presentation” (p=0.037, Δ=-0.132), “interdisciplinary knowledge” (p=0.027, Δ=-0.126), “scope of content” (p=0.025, Δ=-0.144), “competence of presenters” (p=0.016, Δ=-0.125), “presentation of conflicts of interest” (p=0.004, Δ=-0.190), and “clinical practical skills” (p=0.002, Δ=-0.148). Within A-PED, females rated “processing/mentioning of learning objectives” higher than males (p=0.033, Δ=0.230). In S-PED, males gave marginally higher scores for “recommendation of the event” (p=0.017, Δ=-0.091). Location influenced A-PED (“communication/social skills”, p=0.034, Δ=-0.257), while specialty affected S-PED self-assessments (e.g., anesthesia vs. trauma, p=0.037, Δ=-0.33). Residents in A-PED rated “selection of contributions” less favorably (p=0.037, Δ=0.514), whereas specialists in S-PED tended to give consistently favorable evaluations (p<0.05, Δ up to 0.176). Workplace differences emerged as well, with university affiliations affecting ratings in both A-PED and S-PED (p<0.05, Δ=±0.333) (see figure 2 (Fig. 2)).

### 3.4. Comparison of adult emergency medicine courses (A-ALS vs. S-ALS)

S-ALS was rated more favorably than A-ALS in “current guidelines” (p=0.001, Δ=0.14), “communication/social skills” (p=0.006, Δ=0.14), “selection of contributions” (p=0.00659, Δ=0.18), “scope of content” (p=0.001, Δ=0.21), “critically reflective presentation” (p=0.00788, Δ=0.14), “own level of competence” (p=0.001, Δ=0.24), “presentation of conflicts of interest” (p=0.019, Δ=0.17), “processing/mentioning of learning objectives” (p=0.004, Δ=0.15) and “quality of working materials” (p=0.028, Δ=0.13). In contrast, A-ALS scored better on “presentation of conflicts of interest” (p=0.019, Δ=0.17) and “event moderation” (p=0.038, Δ=-0.13).

**Figure 3:**
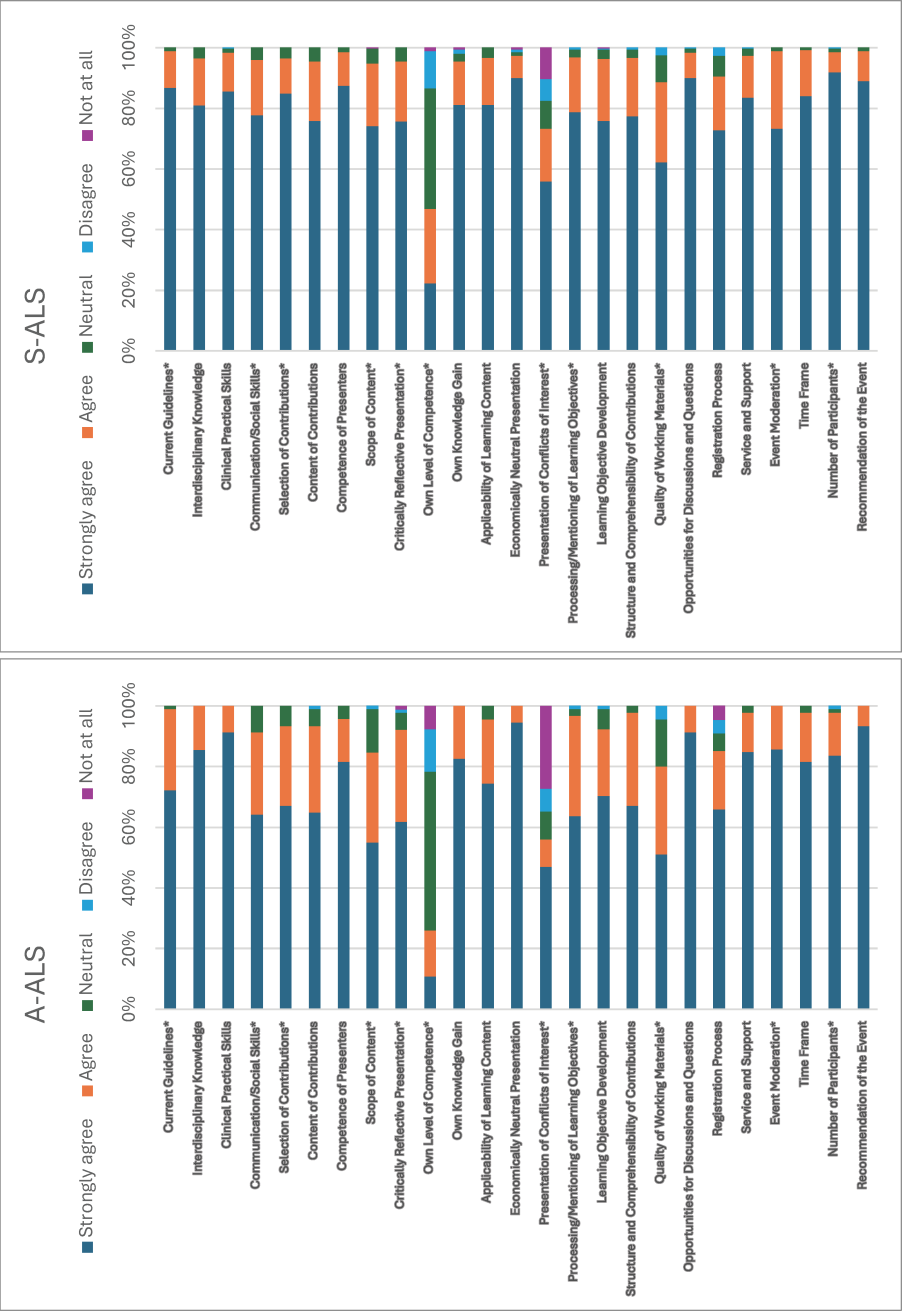
Stacked bar chart showing the percentage distribution of ratings by category for both the accredited adult emergency medicine course (A-ALS) and the in-house (S-ALS). Asterisks (*) mark significant differences based on the U test (p<0.05). Full percentage data is provided in attachment 4.

Location influenced “competence of presenters” in A-ALS (p=0.040, Δ=±0.197) and “opportunities for discussions and questions” (p=0.003, Δ=±0.198) and “registration process” (p=0.012, Δ=±0.256) in S-ALS. Specialty and training stage also mattered in S-ALS, with residents rating some aspects lower (Δ=-0.1 to -0.18) and specialists/non-physicians rating others higher (Δ=0.13 to 0.23, p<0.05). Workplace settings in S-ALS influenced ratings of “communication/social skills,” “learning objective development,” and “quality of working materials” (p<0.05, Δ=-0.15 to -0.19), as well as perceptions of presenter competence and discussion opportunities (p<0.05, Δ=±0.09) (see figure 3 (Fig. 3)).

### 3.5. Free text responses

Across 163 free-text responses, participants frequently praised the courses for their practical orientation, capable instructors, and small group sizes (see attachment 3 ). A-PHEM excelled in practical skill training, scenario diversity, and clear instruction but required earlier material distribution, more specialized skills, and improved logistics. S-PHEM stood out for realistic scenarios, engaging debriefings, and a supportive atmosphere yet needed better technical setups, standardized structures, and enhanced e-learning. In pediatrics, A-PED offered effective debriefing, practical relevance, and stable team structures, though alignment with current guidelines, smaller groups, and clearer scenarios were suggested. S-PED was recognized for its high practicality, small group sizes, and interdisciplinary approach but called for a more balanced theory-to-practice ratio, larger break areas, and clearer preparation materials. Finally, A-ALS combined a structured design with positive instructor engagement, while S-ALS provided hands-on training and small groups – both required refined logistics, earlier materials, and extended or more diverse scenarios. Detailed summaries of these recurring themes and feedback can be found in the supplementary material (see attachment 4 ).

## 4. Discussion

Comparisons of in-house (S-PHEM, S-PED, S-ALS) and society-accredited (A-PHEM, A-PED, A-ALS) SBME courses generally showed that accredited formats excelled in guideline adherence, organizational structure, and critical reflection – especially in prehospital and pediatric contexts. However, in the ALS domain, the in-house course was rated more favorably on “current guidelines” and several other aspects, while the accredited course received higher scores for event moderation. Demographic analyses revealed variations linked to gender, specialty, and workplace, hinting at specific needs or barriers in knowledge transfer; for example, gender-specific differences in communication and moderation styles may require targeted teaching strategies. In their free-text responses, participants praised the courses for their practical orientation, knowledgeable instructors, and small group sizes, yet recommended longer course durations, earlier material distribution, closer guideline alignment, and logistical refinements.

### Does it really matter if the course is certified or not?

Whether a course is certified can significantly impact its quality, standardisation, and recognition within the medical community [21], [22]. Although accredited courses, as seen in this study – long recognized in the literature for their adherence to guidelines and organizational consistency – exhibited notable strengths, they also showed drawbacks [20], [23], [24], [25]. Participant feedback noted rigidity in accredited course structures, limited adaptability to learners’ needs, and occasional outdated content. In contrast, in-house formats offered flexible, interactive learning with tailored content and dynamic discussions. This adaptability – combined with guideline adherence – suggests refining both formats to capitalize on their strengths. In-house designs enable scenario customization, active engagement, and practical reflections, which participants especially valued for interdisciplinary collaboration, event moderation, and instructor involvement. 

### Impact of curriculum and structure vs other factors like instructor competency

The interplay between curriculum/structure and instructor competency is crucial in SBME effectiveness. While curriculum and structure lay the foundation for learning objectives and content, the instructor is essential for engagement, guiding discussions, and feedback [26]. A well-designed curriculum aligns with educational goals and supports active learning through simulations and hands-on activities [27]. However, its effectiveness can be enhanced or limited by the instructor’s ability to adapt teaching methods, foster a supportive environment, and offer individualized guidance. Therefore, even though curriculum and structure provide the framework for learning outcomes, the instructor’s expertise, communication skills, and teaching approach fundamentally shape the educational experience and learner engagement [13], [14], [28]. 

The role and competence of instructors seem to represent a significant factor influencing the success of SBME courses. While instructors in certified courses undergo rigorous training programs and formal certification processes, in-house courses often provide a more flexible structure, allowing experienced local experts to deliver content tailored to specific needs practically. Our findings indicate that the perception of instructor competence significantly contributes to participant satisfaction, particularly in moderation, discussion, and hands-on guidance. Future studies should further investigate the impact of instructor qualifications and experience on learning quality to identify additional opportunities for optimization. Ultimately, a synergistic relationship between curriculum design and instructor competency is essential for maximising the impact of SBME courses on knowledge acquisition, skill development, and clinical practice readiness.

### Implications for medical education 

Our findings highlight the importance of a differentiated approach to designing SBME courses. While certified courses offer advantages in standardized content and structure, in-house courses excel through flexibility and practice-oriented customisation. These insights can serve as a foundation for developing a hybrid course model that integrates the strengths of both formats and specifically addresses learners’ needs. This approach can ensure that medical training adheres to high standards and remains adaptive and participant-centred, fostering a more robust learning environment [29]. For healthcare professionals, choosing a mix of both accredited and in-house designed courses could maximise learning outcomes, preparing them better for clinical practice. 

### Study limitations

Our study is limited by its single-institution scope, which may reduce generalizability. Because we primarily rely on self-reported data, response or social desirability bias cannot be ruled out. Although we compare course structures, we do not control for variations in instructor competence or teaching style, which can significantly influence learning outcomes. While our analysis highlighted these themes effectively, a more systematic content analysis, such as Mayring's methodology, could offer a deeper understanding of qualitative patterns and is recommended for future research [30]. We also do not include longitudinal data to assess the long-term retention of skills and knowledge or the impact on clinical practice. Detailed data on participants' expertise, such as years of clinical experience or prior simulation training, were not systematically collected in our study. We acknowledge these limitation and recommend addressing these aspects in future research to enable a more nuanced analysis of group outcomes.

## 5. Conclusion

Both in-house and society-accredited SBME courses exhibit distinct strengths and areas requiring further refinement. In prehospital (PHEM) and pediatric (PED) courses, society-accredited formats generally demonstrated stronger guideline adherence and organizational structure, whereas in the adult (ALS) domain, the in-house course achieved higher ratings in several key dimensions, despite being shorter (10h vs 20h). These findings emphasize the potential value of integrating best practices from both approaches to enhance medical education and training outcomes. Future research should explore the long-term impact of instructor qualifications, examine demographic factors (e.g., gender) in shaping course evaluations, and conduct longitudinal studies on the sustainability of learning gains. Ultimately, developing a hybrid course format that leverages the advantages of both in-house and accredited designs may further optimize SBME programs.

## Key points

- Society- accredited SBME courses, like those from the European Resuscitation Council and American Heart Association, provide consistency, quality, and international recognition, but may limit opportunities for individualized learning.
- In-house designed formats excell in areas like content scope and communication.
- Both in-house designed and society-accredited courses have distinct strengths and areas for improvement.

## Abbreviations

- AHA: American Heart Association
- A-ALS: ERC Advanced Life Support simulation format
- S-PED: In-house paediatric emergency course: Bärenkind – Berliner Ärzte retten Kinder – Emergency Netzwerk für das Kind
- BeST: Berlin Simulation & Training Center
- CAPCE: Commission on Accreditation for Prehospital Continuing Education
- CME: Continuing Medical Education
- A-PED: ERC European Paediatric Advanced Life Support simulation format
- ERC: European Resuscitation Council
- S-PHEM: In-house prehospital emergency medicine simulation course - Notarztsimulation
- A-PHEM: Prehospital Trauma Life Support accredited by Commission on Accreditation for Prehospital Continuing Education
- S-ALS: In-house Advanced Life Support format
- SBME: Simulation-Based Medical Education

## Notes

### Conference presentation

Data was partially presented at ERC Resuscitation 2023 in Barcelona, Spain.

### Authors’ contributions:

- IA: Conception of work, drafting of the manuscript, and data analysis.
- JB: Data acquisition and substantive revision of the manuscript.
- EK: Data acquisition and substantive revision of the manuscript.
- JBE: Data analysis, data interpretation, and substantive revision of the manuscript.
- TS: Conception of work and substantive revision of the manuscript.
- The authors JBE and TS share the last-authorship.

### Authors’ ORCIDs

- Igor Abramovich: [0000-0002-1760-6223]
- Eva Kornemann: [0009-0006-3871-1184]
- Joana Berger-Estilita: [0000-0002-8695-4264]
- Torsten Schröder: [0000-0002-0388-1558]

## Acknowledgements

We thank all past and present members of the Berlin Training and Simulation Center (BeST) who supported this study.

## Competing interests

The authors declare that they have no competing interests. 

## Supplementary Material

Translation of the evaluation form of the Berlin Medical Association

Overview of primary learning objectives, focus and key goals of each course in the study

Free-text comments

Percentage distribution

## Figures and Tables

**Table 1:**
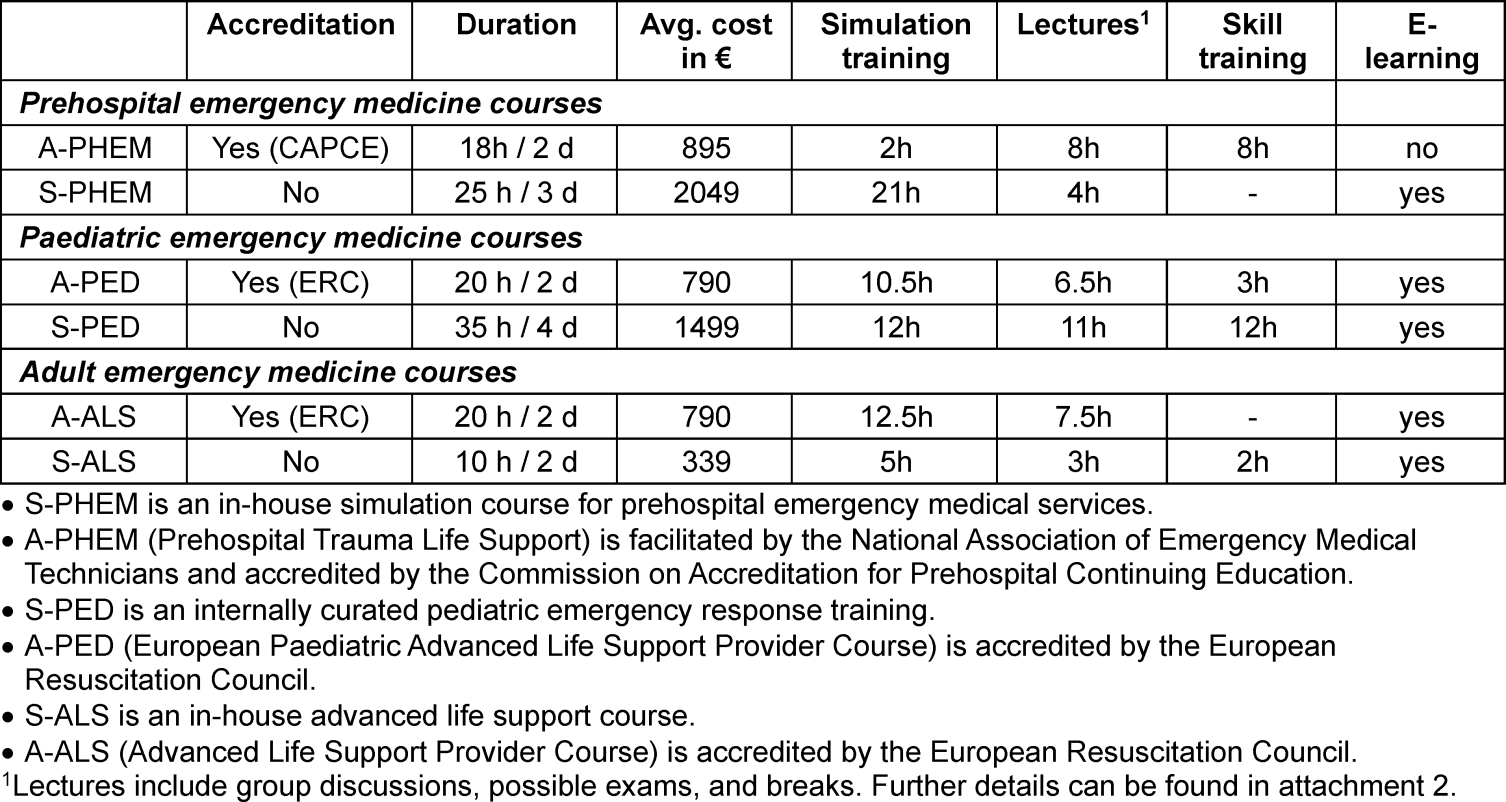
This table compares the scope, financial considerations, and subject matter of in-house courses* vs. those certified by external accrediting societies

**Table 2:**
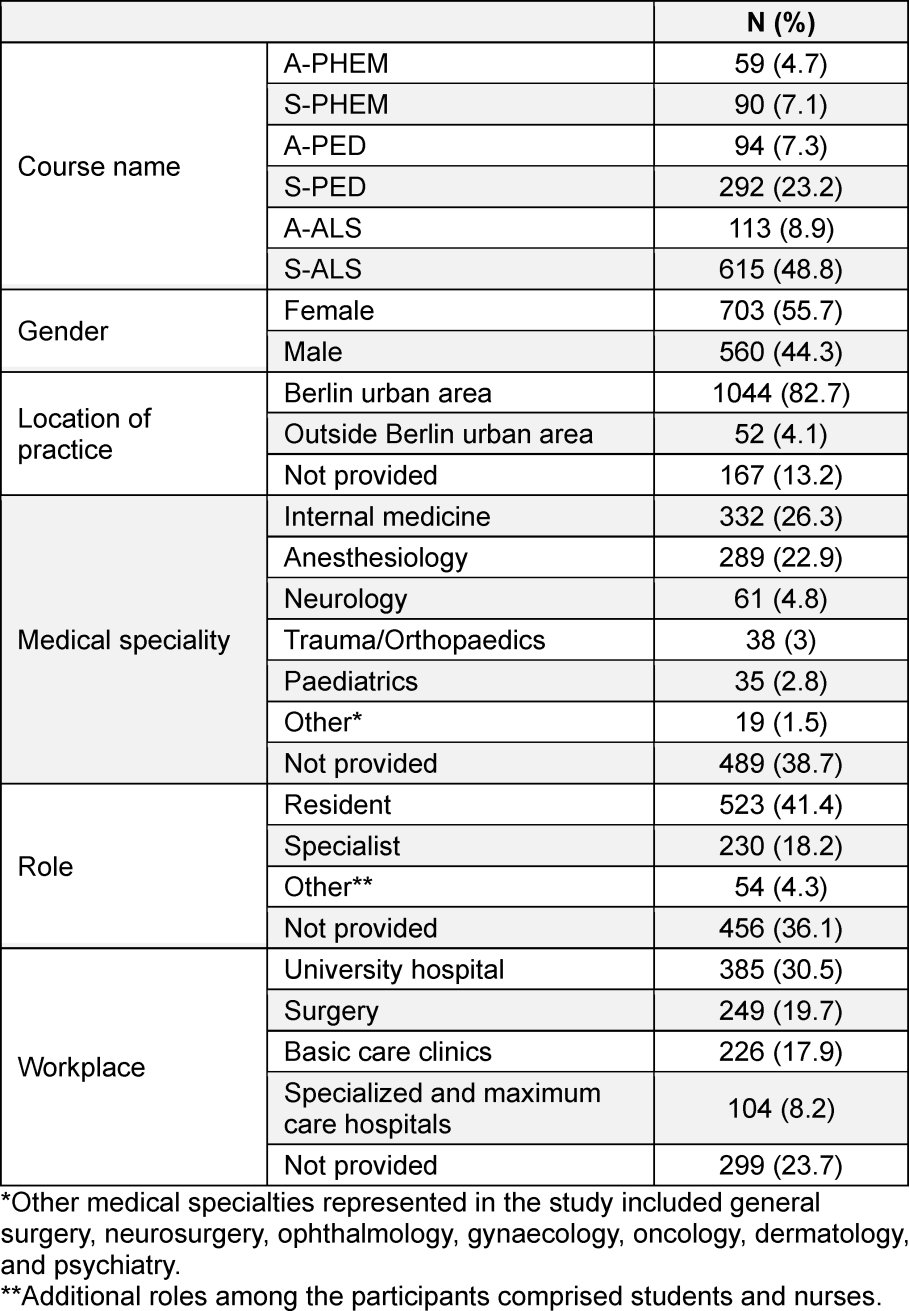
Number of course participants per course type and demographic data

**Table 3:**
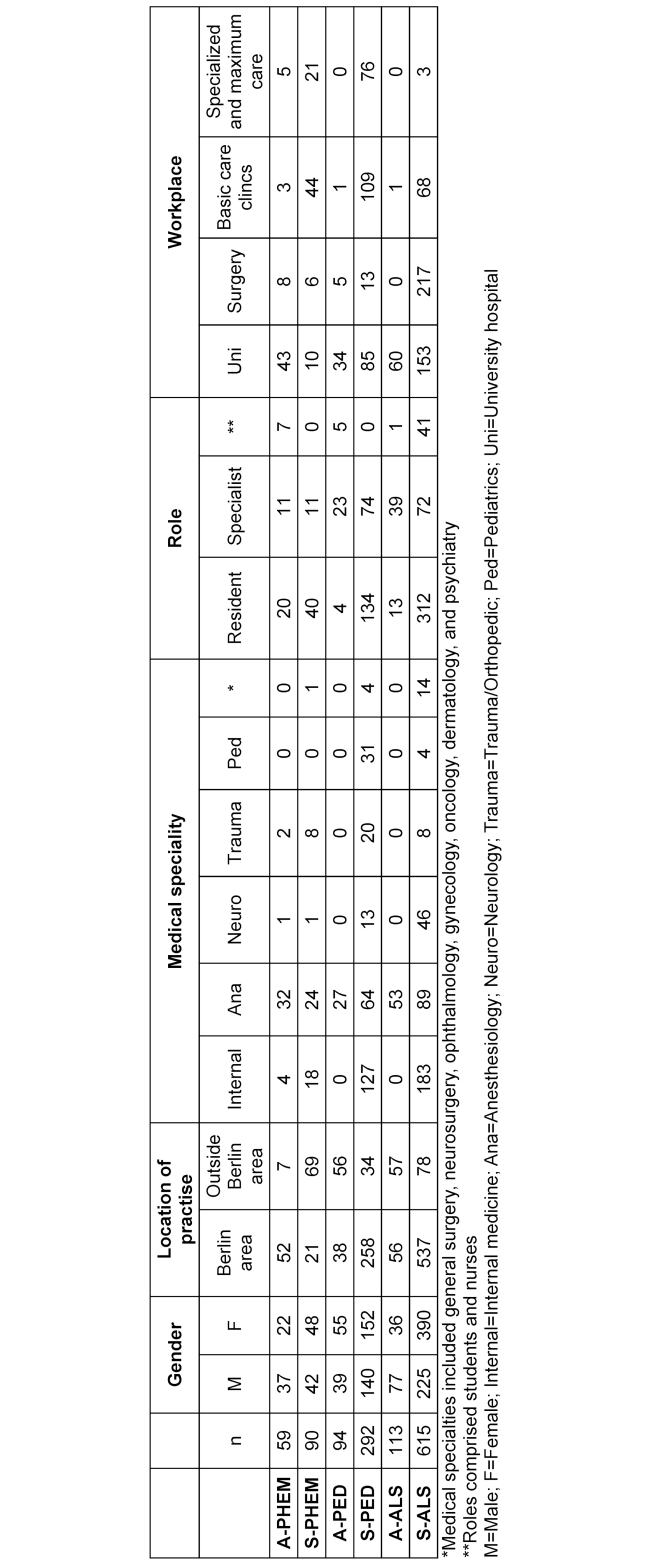
Detailed breakdown of participant demographics of participants who provide information in the above categories, not including not provided answers
