# Supplementary material for: In-house designed simulation courses versus society-accredited designs by international societies: A comparative analysis
Source: GMS J Med Educ. 2025 Jun 16;42(3):Doc32. doi: 10.3205/zma001756 (PMC12286873; doi:10.3205/zma001756)
Supplement: Translation of the evaluation form of the Berlin Medical Association [file JME-42-32-s-001.pdf]

Attachment 1: Translation of the evaluation form of the Berlin Medical Association

Event:

Date:

VNR:

This evaluation form template is provided by the Berlin Medical Association.

Content	Strongly agree -				not at all -	N/A
The event conveyed current scientific information Findings and/or guidelines.	☐	☐	☐	☐	☐	☐
The event conveyed interdisciplinary and interdisciplinary knowledge.	☐	☐	☐	☐	☐	☐
The event imparted clinical-practical skills.	☐	☐	☐	☐	☐	☐
The event conveyed communicative and social competencies.	☐	☐	☐	☐	☐	☐
The selection of contributions covered the relevant aspects of the topic(s).	☐	☐	☐	☐	☐	☐
The content of the contributions was well coordinated.	☐	☐	☐	☐	☐	☐
The speakers were for the topics of their Contributions competent	☐	☐	☐	☐	☐	☐
The content of the topics was appropriate shown.	☐	☐	☐	☐	☐	☐

Attachment 1 to Abramovich I, Beilstein J, Kornemann E, Berger-Estilita J, Schröder T. In-house designed simulation courses versus society-accredited designs by international societies: A comparative analysis. GMS J Med Educ. 2025;42(3):Doc32. DOI: 10.3205/zma001756

The content of the contributions became critically reflective shown	☐	☐	☐	☐	☐	☐
My competencies in the event topics were already good.	☐	☐	☐	☐	☐	☐
I was able to expand my skills.	☐	☐	☐	☐	☐	☐
What I learned in the event I can use in mine implement activity.	☐	☐	☐	☐	☐	☐
The presented contents of the event were free of economic interests..	☐	☐	☐	☐	☐	☐
The speakers presented whether a conflict of interest existed or not.	☐	☐	☐	☐	☐	☐
Content delivery						
Learning objectives (knowledge and skills that are event should be conveyed) were mentioned.	☐	☐	☐	☐	☐	☐
During the event I have learning outcomes myself or worked out in a group.	☐	☐	☐	☐	☐	☐
The contributions were well structured and easy to understand.	☐	☐	☐	☐	☐	☐
The working materials provided were appropriate and well designed.	☐	☐	☐	☐	☐	☐
It was possible to ask questions and discuss the topic discuss.	☐	☐	☐	☐	☐	☐

This evaluation form template is provided by the Berlin Chamber of Doctors.

Event:

Date:

VNR:

	Strongly agree -			not at all -		N/A
**Organisation**						
The registration process went smoothly.	☐	☐	☐	☐	☐	☐
The service and support during the event was good.	☐	☐	☐	☐	☐	☐
The entire event was well moderated.	☐	☐	☐	☐	☐	☐
The schedule was met.	☐	☐	☐	☐	☐	☐
The number of participants was appropriate for the event.	☐	☐	☐	☐	☐	☐
**Would you like to add anything else?**						
What I particularly liked about this event was:						
I would have liked to have changed or improved this event:						

Conclusion		
I can recommend attending this event.	☐ ☐ ☐ ☐ ☐	☐

I became aware of this event through:

☐ E-Mail/Flyer from the Organiser

☐ Personal recommendation

☐ Magazine, e.g. specialist journal

☐ Training calendar or Internet

☐ Other:

.... I

am:

☐ Medical Student

☐ Trainee/Resident

☐ Specialist

☐ Other professional group: ....

I am currently working completely or mainly:

☐ in the inpatient area

☐ in the outpatient area

☐ public health services

☐ in …………………………………………………………….

☐ currently not employed.

Thank you for your support!

This evaluation form template is provided by the Berlin Chamber of Doctors.

Attachment 1 to Abramovich I, Beilstein J, Kornemann E, Berger-Estilita J, Schröder T. In-house designed simulation courses versus society-accredited designs by international societies: A comparative analysis. GMS J Med Educ. 2025;42(3):Doc32. DOI: 10.3205/zma001756
