# Supplementary material for: In-house designed simulation courses versus society-accredited designs by international societies: A comparative analysis
Source: GMS J Med Educ. 2025 Jun 16;42(3):Doc32. doi: 10.3205/zma001756 (PMC12286873; doi:10.3205/zma001756)
Supplement: Overview of primary learning objectives, focus and key goals of each course in the study [file JME-42-32-s-002.pdf]

Attachment 2: Overview of primary learning objectives, focus and key goals of each course in the study

Course format	Primary learning objectives	Focus and key goals
**A-PHEM** *(Accredited Prehospital Emergency Medicine Course)*	- Application of current prehospital emergency guidelines.<br>- Effective teamwork in high-stress scenarios.	Standardized training with a focus on adherence to international prehospital care guidelines and protocols
**S-PHEM** *(In-House Prehospital Emergency Medicine Course)*	- Adaptation of prehospital interventions to local protocols.<br>- Emphasis on scenariobased training.	Flexible approach tailored to institution-specific requirements and participant needs
**A-PED** *(Accredited Pediatric Emergency Medicine Course)*	- Mastery of pediatric life support protocols.<br>- Development of effective communication skills in pediatric emergencies.	Structured, protocol-driven pediatric emergency response training
**S-PED** *(In-House Pediatric Emergency Medicine Course)*	- Customized approaches to pediatric emergencies.<br>- Focus on hands-on, scenario-driven learning.	Adaptable to specific institutional and learner needs, with interactive case-based scenarios
**A-ALS** *(Accredited Advanced Life Support Course)*	- Proficiency in advanced life support techniques.<br>- Adherence to standardized resuscitation protocols.	Rigorous focus on international resuscitation standards and teamwork
**S-ALS** *(In-House Advanced Life Support Course)*	- Scenario-based application of resuscitation skills.<br>- Adaptability to local clinical workflows.	Interactive and flexible training with emphasis on team dynamics and practical skills

Attachment 2 to Abramovich I, Beilstein J, Kornemann E, Berger-Estilita J, Schröder T. In-house designed simulation courses versus society-accredited designs by international societies: A comparative analysis. GMS J Med Educ. 2025;42(3):Doc32. DOI: 10.3205/zma001756
