# Supplementary material for: In-house designed simulation courses versus society-accredited designs by international societies: A comparative analysis
Source: GMS J Med Educ. 2025 Jun 16;42(3):Doc32. doi: 10.3205/zma001756 (PMC12286873; doi:10.3205/zma001756)
Supplement: Free-text comments [file JME-42-32-s-003.pdf]

Attachment 3: Free-text comments

Each course type demonstrates unique strengths and areas for improvement, reflecting the differing priorities and structures of in-house and accredited course designs. These insights were derived from 163 free-text comments (99 positive and 64 suggestions for improvement) and highlight actionable areas for course optimization.

Course type	Recurring positive themes	Recurring suggestions for improvement
**A-PHEM** *(Accredited Prehospital Emergency Medicine Course)*	- Practical skill training<br>- Scenario diversity<br>- Clear instructional methods<br>- Instructor responsiveness	- Earlier distribution of course materials<br>- More focus on specific skills (e.g., patient extraction from vehicles)<br>- Improved course logistics
**S-PHEM** *(In-House Prehospital Emergency Medicine Course)*	- Realistic scenarios<br>- Engaging debriefing sessions<br>- Competence of instructors<br>- Practical relevance<br>- Supportive team atmosphere	- Improved technical setup (e.g., sound, equipment)<br>- More standardized structure<br>- Enhanced E-learning materials<br>- Better facilities
**A-PED** *(Accredited Pediatric Emergency Medicine Course)*	- Effective debriefing sessions<br>- Practical relevance<br>- Constant team structure<br>- Ample training materials<br>- Positive team dynamic	- Updated alignment with current guidelines<br>- Smaller group sizes<br>- Improved organization of pre-course preparation<br>- Clearer scenario alignment
**S-PED** *(In-House Pediatric Emergency Medicine Course)*	- High practical component<br>- Small group sizes<br>- Instructor expertise<br>- Interdisciplinary approach<br>- Positive team atmosphere	- More balanced theory-to-practice ratio<br>- Larger break areas<br>- Earlier access to E-learning content<br>- Clearer preparation materials
**A-ALS** *(Accredited Advanced Life Support Course)*	- Structured course design<br>- Practical relevance<br>- Positive instructor engagement<br>- Team-oriented atmosphere<br>- Effective feedback sessions	- Shorter lecture durations<br>- Improved logistical communication<br>- Earlier access to course materials<br>- More peri-arrest scenarios
**S-ALS** *(In-House Advanced Life Support Course)*	- Practical exercises and hands-on training<br>- Small group sizes<br>- Competence and enthusiasm of instructors<br>- Interactive scenarios<br>- Positive learning atmosphere	- Longer course duration<br>- More case-based scenarios<br>- Improved technical setup<br>- Enhanced clarity in registration and materials access
