# Supplementary material for: In-house designed simulation courses versus society-accredited designs by international societies: A comparative analysis
Source: GMS J Med Educ. 2025 Jun 16;42(3):Doc32. doi: 10.3205/zma001756 (PMC12286873; doi:10.3205/zma001756)
Supplement: Percentage distribution [file JME-42-32-s-004.pdf]

Attachment 4: Percentage distribution

A-PHEM – percentage distribution

Category	Strongly agree	Agree	Neutral	Disagree	Not at all
Current guidelines*	96.30%	3.70%	0.00%	0.00%	0.00%
Interdisciplinary knowledge	88.90%	7.40%	3.70%	0.00%	0.00%
Clinical practical skills	88.90%	9.30%	1.90%	0.00%	0.00%
Communication/social skills*	85.20%	13.00%	1.90%	0.00%	0.00%
Selection of contributions*	87.00%	11.10%	1.90%	0.00%	0.00%
Content of contributions	92.60%	7.40%	0.00%	0.00%	0.00%
Competence of presenters	90.70%	9.30%	0.00%	0.00%	0.00%
Scope of content*	85.20%	13.00%	1.90%	0.00%	0.00%
Critically reflective presentation	88.70%	9.40%	1.90%	0.00%	0.00%
Own level of competence*	24.10%	31.50%	37.00%	7.40%	0.00%
Own knowledge gain	75.90%	22.20%	1.90%	0.00%	0.00%
Applicability of learning content	83.30%	13.00%	3.70%	0.00%	0.00%
Economically neutral presentation	94.40%	5.60%	0.00%	0.00%	0.00%
Presentation of conflicts of interest*	85.70%	6.10%	6.10%	2.00%	0.00%
Processing/mentioning of learning objectives*	79.60%	20.40%	0.00%	0.00%	0.00%
Learning objective development	87.00%	11.10%	1.90%	0.00%	0.00%
Structure and comprehensibility of contributions	90.70%	7.40%	1.90%	0.00%	0.00%
Quality of working materials	71.70%	22.60%	5.70%	0.00%	0.00%
Opportunities for discussions and questions	100.00%	0.00%	0.00%	0.00%	0.00%
Registration process*	75.90%	13.00%	7.40%	1.90%	1.90%
Service and support	88.90%	7.40%	3.70%	0.00%	0.00%
Event moderation	98.10%	1.90%	0.00%	0.00%	0.00%
Time frame*	98.10%	1.90%	0.00%	0.00%	0.00%
Number of participants	98.10%	1.90%	0.00%	0.00%	0.00%
Recommendation of the event	90.60%	9.40%	0.00%	0.00%	0.00%

S-PHEM – percentage distribution

Category	Strongly agree	Agree	Neutral	Disagree	Not at all
Current guidelines*	80.80%	19.20%	0.00%	0.00%	0.00%
Interdisciplinary knowledge	77.00%	18.90%	4.10%	0.00%	0.00%
Clinical practical skills	91.90%	6.80%	1.40%	0.00%	0.00%
Communication/social skills*	68.90%	24.30%	6.80%	0.00%	0.00%
Selection of contributions*	71.20%	20.50%	8.20%	0.00%	0.00%
Content of contributions	73.00%	24.30%	2.70%	0.00%	0.00%
Competence of presenters	93.20%	5.40%	1.40%	0.00%	0.00%
Scope of content*	63.50%	31.10%	5.40%	0.00%	0.00%
Critically reflective presentation	75.00%	22.20%	2.80%	0.00%	0.00%
Own Level of competence*	13.70%	23.30%	46.60%	15.10%	1.40%
Own knowledge gain	81.10%	13.50%	5.40%	0.00%	0.00%
Applicability of learning content	67.60%	23.00%	8.10%	1.40%	0.00%
Economically neutral presentation	94.50%	2.70%	1.40%	0.00%	1.40%
Presentation of conflicts of interest*	54.40%	8.80%	19.30%	3.50%	14.00%
Processing/mentioning of learning objectives*	67.60%	24.30%	8.10%	0.00%	0.00%
Learning objective development	75.70%	23.00%	1.40%	0.00%	0.00%
Structure and comprehensibility of contributions	82.40%	17.60%	0.00%	0.00%	0.00%
Quality of working materials	64.40%	21.90%	11.00%	2.70%	0.00%
Opportunities for discussions and questions	95.90%	4.10%	0.00%	0.00%	0.00%
Registration process*	59.70%	13.90%	18.10%	6.90%	1.40%
Service and support	82.20%	12.30%	4.10%	1.40%	0.00%
Event moderation	93.10%	6.90%	0.00%	0.00%	0.00%
Time frame*	87.70%	12.30%	0.00%	0.00%	0.00%
Number of participants	90.30%	6.90%	0.00%	2.80%	0.00%
Recommendation of the event	88.60%	11.40%	0.00%	0.00%	0.00%

A-PED – percentage distribution

Category	Strongly agree	Agree	Neutral	Disagree	Not at all
Current guidelines	96.67%	3.33%	0.00%	0.00%	0.00%
Interdisciplinary knowledge*	91.53%	6.78%	1.69%	0.00%	0.00%
Clinical practical skills*	100.00%	0.00%	0.00%	0.00%	0.00%
Communication/social skills	78.33%	18.33%	3.33%	0.00%	0.00%
Selection of contributions	78.33%	21.67%	0.00%	0.00%	0.00%
Content of contributions	76.67%	20.00%	3.33%	0.00%	0.00%
Competence of presenters*	95.00%	5.00%	0.00%	0.00%	0.00%
Scope of content*	85.00%	13.33%	1.67%	0.00%	0.00%
Critically reflective presentation*	85.00%	15.00%	0.00%	0.00%	0.00%
Own level of competence	38.33%	15.00%	36.67%	10.00%	0.00%
Own knowledge gain	88.33%	8.33%	3.33%	0.00%	0.00%
Applicability of learning content	76.67%	18.33%	5.00%	0.00%	0.00%
Economically neutral presentation	96.67%	3.33%	0.00%	0.00%	0.00%
Presentation of conflicts of interest*	89.66%	5.17%	3.45%	0.00%	1.72%
Processing/mentioning of learning objectives	80.00%	18.33%	1.67%	0.00%	0.00%
Learning objective development	76.67%	20.00%	3.33%	0.00%	0.00%
Structure and comprehensibility of contributions	83.33%	15.00%	1.67%	0.00%	0.00%
Quality of working materials	72.88%	20.34%	6.78%	0.00%	0.00%
Opportunities for discussions and questions	95.00%	5.00%	0.00%	0.00%	0.00%
Registration process	84.75%	13.56%	1.69%	0.00%	0.00%
Service and support	86.67%	11.67%	1.67%	0.00%	0.00%
Event moderation	96.67%	3.33%	0.00%	0.00%	0.00%
Time frame	89.83%	10.17%	0.00%	0.00%	0.00%
Number of participants	98.31%	1.69%	0.00%	0.00%	0.00%
Recommendation of the event	96.49%	3.51%	0.00%	0.00%	0.00%

S-PED – percentage distribution

Category	Strongly agree	Agree	Neutral	Disagree	Not at all
Current guidelines	88.26%	10.00%	1.74%	0.00%	0.00%
Interdisciplinary knowledge*	78.70%	19.57%	1.74%	0.00%	0.00%
Clinical practical skills*	85.15%	12.66%	1.75%	0.44%	0.00%
Communication/social skills	81.30%	14.78%	3.48%	0.43%	0.00%
Selection of contributions	82.63%	15.96%	0.94%	0.47%	0.00%
Content of contributions	80.28%	19.25%	0.47%	0.00%	0.00%
Competence of presenters*	82.61%	16.09%	0.87%	0.43%	0.00%
Scope of content*	70.87%	24.35%	4.78%	0.00%	0.00%
Critically reflective presentation*	72.69%	21.59%	4.85%	0.44%	0.44%
Own level of competence	32.89%	21.49%	33.33%	10.96%	1.32%
Own knowledge gain	81.30%	15.65%	2.61%	0.00%	0.43%
Applicability of learning content	79.82%	17.11%	3.07%	0.00%	0.00%
Economically neutral presentation	93.72%	4.48%	1.79%	0.00%	0.00%
Presentation of conflicts of interest*	70.87%	14.08%	5.83%	3.40%	5.83%
Processing/mentioning of learning objectives	77.40%	18.75%	3.37%	0.48%	0.00%
Learning objective development	79.61%	15.53%	4.37%	0.49%	0.00%
Structure and comprehensibility of contributions	82.21%	15.87%	1.44%	0.48%	0.00%
Quality of working materials	67.31%	21.15%	10.10%	0.48%	0.96%
Opportunities for discussions and questions	92.79%	4.33%	1.92%	0.96%	0.00%
Registration process	79.71%	13.53%	5.31%	1.45%	0.00%
Service and support	88.89%	10.14%	0.97%	0.00%	0.00%
Event moderation	90.00%	8.95%	1.05%	0.00%	0.00%
Time frame	86.96%	12.56%	0.00%	0.48%	0.00%
Number of participants	91.79%	6.28%	1.45%	0.00%	0.48%
Recommendation of the event	91.67%	7.84%	0.49%	0.00%	0.00%

A-ALS – percentage distribution

Category	Strongly agree	Agree	Neutral	Disagree	Not at all
Current guidelines*	72.22%	26.67%	1.11%	0.00%	0.00%
Interdisciplinary knowledge	85.56%	14.44%	0.00%	0.00%	0.00%
Clinical practical skills	91.30%	8.70%	0.00%	0.00%	0.00%
Communication/social skills*	64.13%	27.17%	8.70%	0.00%	0.00%
Selection of contributions*	67.03%	26.37%	6.59%	0.00%	0.00%
Content of contributions	64.84%	28.57%	5.49%	1.10%	0.00%
Competence of presenters	81.52%	14.13%	4.35%	0.00%	0.00%
Scope of content*	54.95%	29.67%	14.29%	1.10%	0.00%
Critically reflective presentation*	61.80%	30.34%	5.62%	1.12%	1.12%
Own level of competence*	10.87%	15.22%	52.17%	14.13%	7.61%
Own knowledge gain	82.61%	17.39%	0.00%	0.00%	0.00%
Applicability of learning content	74.44%	21.11%	4.44%	0.00%	0.00%
Economically neutral presentation	94.51%	5.49%	0.00%	0.00%	0.00%
Presentation of conflicts of interest*	46.97%	9.09%	9.09%	7.58%	27.27%
Processing/mentioning of learning objectives*	63.74%	32.97%	2.20%	1.10%	0.00%
Learning objective development	70.33%	21.98%	6.59%	1.10%	0.00%
Structure and comprehensibility of contributions	67.03%	30.77%	2.20%	0.00%	0.00%
Quality of working materials*	51.11%	28.89%	15.56%	4.44%	0.00%
Opportunities for discussions and questions	91.30%	8.70%	0.00%	0.00%	0.00%
Registration process	65.91%	19.32%	5.68%	4.55%	4.55%
Service and support	84.78%	13.04%	2.17%	0.00%	0.00%
Event moderation*	85.71%	14.29%	0.00%	0.00%	0.00%
Time frame	81.52%	16.30%	2.17%	0.00%	0.00%
Number of participants*	83.70%	14.13%	1.09%	1.09%	0.00%
Recommendation of the event	93.33%	6.67%	0.00%	0.00%	0.00%

S-ALS – percentage distribution

**Category**	**Strongly agree**	**Agree**	**Neutral**	**Disagree**	**Not at all**
Current guidelines*	86.78%	12.07%	1.15%	0.00%	0.00%
Interdisciplinary knowledge	81.03%	15.52%	3.45%	0.00%	0.00%
Clinical practical skills	85.63%	12.64%	1.44%	0.29%	0.00%
Communication/social skills*	77.71%	18.29%	4.00%	0.00%	0.00%
Selection of contributions*	84.88%	11.63%	3.49%	0.00%	0.00%
Content of contributions	75.86%	19.54%	4.60%	0.00%	0.00%
Competence of presenters	87.39%	11.17%	1.43%	0.00%	0.00%
Scope of content*	74.14%	20.69%	4.89%	0.00%	0.29%
Critically reflective presentation*	75.72%	19.65%	4.62%	0.00%	0.00%
Own level of competence*	22.29%	24.57%	39.71%	12.29%	1.14%
Own knowledge gain	81.14%	14.29%	2.57%	1.43%	0.57%
Applicability of learning content	81.14%	15.43%	3.43%	0.00%	0.00%
Economically neutral presentation	89.91%	7.42%	1.19%	0.89%	0.59%
Presentation of conflicts of interest*	55.87%	17.44%	9.25%	7.12%	10.32%
Processing/mentioning of learning objectives*	78.67%	18.16%	2.59%	0.58%	0.00%
Learning objective development	75.87%	20.35%	3.20%	0.29%	0.29%
Structure and comprehensibility of contributions	77.43%	19.14%	2.86%	0.57%	0.00%
Quality of working materials*	62.29%	26.29%	8.86%	2.57%	0.00%
Opportunities for discussions and questions	89.97%	8.31%	1.43%	0.29%	0.00%
Registration process	72.83%	17.63%	6.94%	2.60%	0.00%
Service and support	83.57%	13.83%	2.31%	0.29%	0.00%
Event moderation*	73.26%	25.58%	1.16%	0.00%	0.00%
Time frame	84.10%	15.03%	0.87%	0.00%	0.00%
Number of participants*	91.88%	6.67%	1.16%	0.29%	0.00%
Recommendation of the event	88.89%	9.91%	1.20%	0.00%	0.00%
